# The Value of OCT and OCTA as Potential Biomarkers for Preclinical Alzheimer’s Disease: A Review Study

**DOI:** 10.3390/life11070712

**Published:** 2021-07-19

**Authors:** Inés López-Cuenca, Elena Salobrar-García, Lorena Elvira-Hurtado, José A. Fernández-Albarral, Lidia Sánchez-Puebla, Juan J. Salazar, José M. Ramírez, Ana I. Ramírez, Rosa de Hoz

**Affiliations:** 1 Ramón Castroviejo Eye Research Institute, Complutense University of Madrid, IdISSC, 28040 Madrid, Spain; inelopez@ucm.es (I.L.-C.); elenasalobrar@med.ucm.es (E.S.-G.); marelvir@ucm.es (L.E.-H.); joseaf08@ucm.es (J.A.F.-A.); lidsan02@ucm.es (L.S.-P.); jjsalazar@med.ucm.es (J.J.S.); ramirezs@med.ucm.es (J.M.R.); 2 OFTARED-ISCIII, 28029 Madrid, Spain; 3 Department of Immunology, Ophthalomolgy and ENT, Faculty of Optics and Optometry, Complutense University of Madrid, 28037 Madrid, Spain; 4 Department of Immunology, Ophthalomolgy and ENT, School of Medicine, Complutense University of Madrid, 28040 Madrid, Spain

**Keywords:** Alzheimer’s disease, preclinical, optical coherence tomography, optical coherence tomography-angiography, retina, biomarker

## Abstract

Preclinical Alzheimer’s disease (AD) includes cognitively healthy subjects with at least one positive biomarker: reduction in cerebrospinal fluid Aβ_42_ or visualization of cerebral amyloidosis by positron emission tomography imaging. The use of these biomarkers is expensive, invasive, and not always possible. It has been shown that the retinal changes measured by optical coherence tomography (OCT) and OCT-angiography (OCTA) could be biomarkers of AD. Diagnosis in early stages before irreversible AD neurological damage takes place is important for the development of new therapeutic interventions. In this review, we summarize the findings of different published studies using OCT and OCTA in participants with preclinical AD. To date, there have been few studies on this topic and they are methodologically very dissimilar. Moreover, these include only two longitudinal studies. For these reasons, it would be interesting to unify the methodology, make the inclusion criteria more rigorous, and conduct longer longitudinal studies to assess the evolution of these subjects. If the results were consistent across repeated studies with the same methodology, this could provide us with insight into the value of the retinal changes observed by OCT/OCTA as potential reliable, cost-effective, and noninvasive biomarkers of preclinical AD.

## 1. Introduction

Alzheimer’s disease (AD) is a neurodegenerative disease that causes the most common type of dementia in the world [1,2], characterized by a progressive decline in memory, learning, and executive functions [3]. The neuropathological hallmarks of AD are extracellular plaques of amyloid-beta (Aβ), cerebral amyloid angiopathy, and intracellular neurofibrillary tangles of hyperphosphorylated tau located mainly in the cerebral cortex [4,5]. There is still no definitive diagnosis for AD before death; however, the pathophysiology of AD can begin decades before the symptoms and the diagnosis of the disease [6]. Thus, new biomarkers are being searched for to enable diagnosis at the preclinical stage of AD. The concept “asymptomatic at-risk patients” was included in the International Working Group criteria, where it was proposed as a state of preclinical AD. This term includes subjects without clinical symptoms or signs, but with biomarkers of Alzheimer’s pathology [7]. Currently, the two standard biomarkers for the diagnosis of preclinical AD are cerebrospinal fluid (CSF) analysis and Aβ positron emission tomography (PET) imaging. However, both diagnostic techniques have their disadvantages: CSF analysis requires a lumbar puncture, an invasive procedure that requires a specialist, and PET is an invasive technique that is very expensive and not always available [8]. Therefore, the discovery of new early biomarkers that are more accessible, cost-effective, and non-invasive to identify people at high risk of developing AD who are cognitively normal would be highly significant. The diagnosis of AD before irreversible neurological damage occurs is important for the development of new therapeutic interventions [9].

The retina is an extension of the central nervous system (CNS), providing a window through which to observe both neuronal and vascular changes in the brain [10]. The importance of monitoring retinal changes in various neurodegenerative diseases has become evident in the last few decades [11,12,13,14,15,16,17], suggesting that the data collected may be useful as biomarkers for the diagnosis and treatment of these diseases [18]. Ophthalmologists now use in vivo imaging techniques that can detect and quantify findings compatible with the histopathologic changes reported in the retinas of AD patients many years ago [19]. Among these imaging techniques, optical coherence tomography (OCT) is a reliable and non-invasive technique commonly used in ophthalmology practices that allows for the visualization and quantification of the retinal layers. This technique allows the observation of the anatomic features of pathological changes in the retina, optic nerve, and choroidal thickness. In the eye, in addition to neural tissue, the retinal vasculature can be analyzed by obtaining non-invasive optical images using OCT-angiography (OCTA).

Both OCT and OCTA are used to monitor retinal changes in the preclinical stages of AD in order to demonstrate whether these changes could be used as biomarkers for the early diagnosis of AD [20,21,22,23,24].

Future research will focus on understanding the pathophysiological process of dementia based on retinal imaging to determine individuals at high risk of developing dementia and evaluate possible treatments for it [10]. This review analyzes the main findings of OCT and OCTA in the preclinical stage of the disease as well as the value of these diagnostic imaging techniques as a contribution to the early diagnosis of subjects at high risk of developing AD.

## 2. Materials and Methods

### 2.1. Search Strategy

We performed a search of the medical literature using the “MESH” terms in PubMed up to April 2021. The search terms were: “optical coherence tomography”, “optical coherence tomography-angiography”, “Alzheimer’s disease”, “preclinical Alzheimer’s disease”, “presymtomatic Alzheimer’s disease”, and “asymptomatic Alzheimer’s disease” as well as their possible combinations. We found 375 articles with the combinations of terms above-mentioned.

### 2.2. Inclusion and Exclusion Criteria

**Figure 1:**
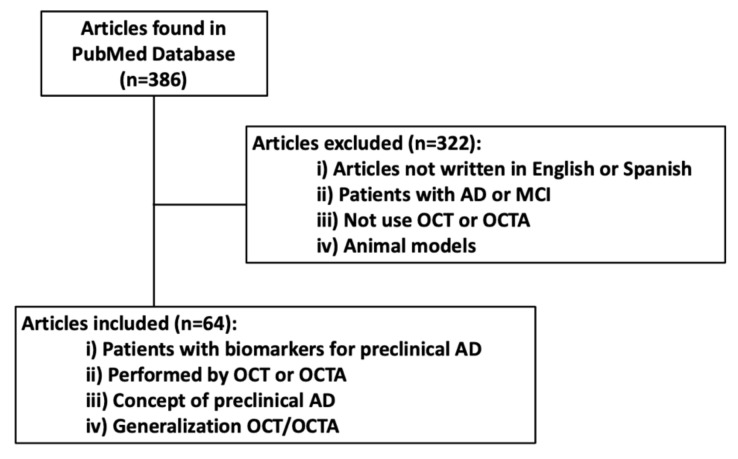
Flow chart of selected articles.

The terms had to be in the title, in the abstract, or in the text of the article. The articles selected were written in English or Spanish and published in the last 10 years. All of them had to relate to the relationship between OCT and OCTA and preclinical AD; we also included articles defining the concept of preclinical Alzheimer’s disease or generalizations about OCT/OCTA as the main topic. Fifty-three articles were considered, while 322 articles did not meet the selection criteria. In Figure 1, we summarize the article selection process.

Inclusion criteria: We considered studies in which participants had one of the two standard biomarkers for the diagnosis of preclinical AD through CSF analysis, and Aβ PET imaging and studies performed with OCT or OCTA.

Exclusion criteria: We excluded studies focused on AD or mild cognitive impairment. We also excluded those that did not use OCT or OCTA in the study. Studies carried out in animal models were also excluded.

## 3. Preclinical AD Definition

The concept of preclinical AD is relatively recent, emerging in the late 20th century [25]. Advances in neuroimaging, cerebrospinal fluid analysis, and other biomarkers have made it possible to detect the pathological process of AD in vivo before clinical manifestations appear in cognitively healthy patients [26].

There are currently two types of biomarkers that determine whether a patient is at asymptomatic risk of developing AD. These are pathophysiological markers and topographical biomarkers. Pathophysiological markers indicate the presence of amyloid pathology (CSF Aβ_42_ or PET amyloid) and Tau pathology (CSF or PET Tau). Topographical markers are volume changes determined by MRI or measured by fluorodeoxyglucose hypometabolism (FDG-PET) in specific brain structures. The biomarkers that are considered diagnostic for defining preclinical AD are the pathophysiological biomarkers CSF Aβ_42_ and amyloid PET. Reference measurement procedures for CSF Aβ_42_ and the first automatic electrochemiluminescence method for the determination of Aβ_42_ in CSF have been established [27,28,29]. Amyloid positivity increases from age 50 onward and correlates very strongly with the presence of the ApoE ɛ4 allele [25]. Furthermore, amyloids in CSF can be the first positive marker [30]; the appearance of Aβ deposits starts 20–30 years before the onset of dementia and is therefore the best marker to determine those at high risk of developing AD [31]. Beta-amyloid deposition can be established using both CSF and PET; CSF has higher sensitivity for detecting amyloid deposition in early stages of AD while amyloid PET may be more specific for detecting individuals who are really on a trajectory of AD. Thus, CSF may be more sensitive for the onset of amyloid deposition, while amyloid PET may be more specific for reporting subjects who will develop AD [25]. For these reasons, one of the two markers is considered sufficient for the diagnosis of preclinical AD.

## 4. Optical Coherence Tomography (OCT)

OCT is a diagnostic technique that provides high-resolution images (micron-precision) of retinal microstructure based on interferometry. This technique is routinely used in ophthalmic diagnostics because it allows quantitative measurements of retinal thickness, choroidal thickness, and retinal sublayer thickness [32,33]. In recent years, the ability of OCT to give high-resolution images has progressively improved; initially, time-domain OCT was rapidly used by neurologists to study retinal nerve fiber layer (RNFL) changes in AD [34]. Subsequently, more advanced spectral domain OCT was used to lengthen the initial findings.

When reviewing the different studies in which retinal damage caused by neurodegenerative disease has been analyzed with OCT, some factors must be taken into account. Numerous OCT systems are available, with segmentation algorithms, scan patterns, and outcome measures varying significantly between different manufacturers [35,36]. Furthermore, even if an OCT from the same manufacturer is used, segmentation algorithms may vary over the course of time and, therefore, measurements obtained with different generations of the same OCT may vary slightly but significantly [36]. Therefore, when making comparisons between different studies, it is very important to take into account the OCT system and scanning parameters used. In addition, most OCT images are of a small area of the retina, with a variation of between 3 mm × 3 mm and 6 mm × 6 mm regions of the optic disc region or central macula. Therefore, most of the retinal tissue is not analyzed with standard OCT imaging protocols. Recently, wide-field systems have allowed 12 mm × 12 mm (or larger) images to be acquired, but they are very expensive, not yet available in all practices, and have a lower resolution. Additionally, all studies reported up to the present time have used bidimensional representations of retinal characteristics; however, these two-dimensional (2D) representations of three-dimensional (3D) biological structures have their limitations. An analysis of 3D versus 2D algorithms would allow retinal findings to be used in the future to study neurodegenerative diseases from a different clinical perspective [37]. Finally, it is noteworthy that the reproducibility of OCT measurements depends on the experience and qualifications of users. In trained hands, the reproducibility of retinal thickness measurements can be very good (intraclass correlation coefficient = 1.0 [38]) and the coefficient of variation very low (between 0.5% [38] and 0.4% [39]).

## 5. Optical Coherence Tomography-Angiography (OCTA)

OCTA is a very recent imaging diagnostic technique based on OCT that is mainly used for the evaluation of the retinal vascularization. This technique is used both for the diagnosis of retinal vascular diseases and for the study of vascularization in pathologies of the optic nerve [40,41,42]. The OCTA information is based on the detection of the movement of hematies inside the retinal capillaries, and the OCTA results are shown as a static map of the retinal capillary density, thus indicating the state of perfusion of the capillaries. OCTA allows the imaging of the retinal vasculature at a near-histological resolution that is superior to imaging with fluorescein angiography, which is an invasive technique [43,44]. In addition, patients with OCTA do not receive intravenous injections of a dye contrast, thus avoiding most of the side effects that occur with conventional angiography (anaphylaxis, nausea, vomiting rash, etc.) [45]. However, OCTA does not provide the information on vascular dynamics that angiography does. Therefore, in those pathologies in which there is an alteration of the blood–retinal barrier with increased vascular permeability, it is preferable to use angiography with different dyes depending on whether we want to analyze the retina (fluorescein) or the choroid (indocyanine green). With OCTA, retinal capillaries are observed in two plexuses, called the superficial and deep retinal plexus. In addition, in the peripapillary region OCTA allows the observation of the peripapillary radial capillaries plexus [43,46]. This technique is suitable for observing changes in retinal microcirculation, which, as discussed above, could correlate with changes in brain microcirculation. The cerebral and retinal vasculature show similarities [47,48]. Both derive from the internal carotid artery and have barrier function (blood–retinal barrier and blood–brain barrier) [49]. In AD patients, it has been suggested that retinal vascular changes share pathogenic mechanisms with those of the cerebral vessels [9,50,51]. Several studies have shown that this correlation exists in patients with AD [23,37].

When analyzing the different studies performed with OCTA, some limitations of this diagnostic technique should be considered. Images obtained by OCTA can have various artifacts, the most frequent being those caused by eye movements relative to the OCT during image acquisition. Motion correction technologies are available, but they also create other artifacts such as stretching, filling, and duplication artifacts. Other artifacts such as loss of signal detection are due to signal blockage by media opacities that affect quantitative OCTA measurements. Flicker artifacts due to eye-tracking technology allow rescanning the area properly. Other problems include segmentation errors, which are more frequent in eyes where the layer limits are irregular. The reproducibility of quantitative measurements can be significantly affected by image decentering, which can be classified according to the distance between the center of the foveal avascular zone (FAZ) and the center of the *en face* image. Finally, projection artifacts are produced when light crossing the superficial vessels is modified by reflection or absorption by hematies and the surrounding tissue. This light is reflected by the retinal pigment epithelium (RPE), creating the false impression that hematies are moving in the RPE vessels and thereby overestimating the perfusion density. Although these projection artifacts are observed very frequently, there are commercially available algorithms for their removal that greatly diminish the impact of these artifacts on the images [37,44,52]. This new modality, due to the need for new equipment and processing techniques, the current limitations of imaging capability, and the rapid advances in both imaging and our understanding of the applicable pathophysiology of the retina and choroid, necessitates a long learning curve. Therefore, the reproducibility of the measurements will depend on the experience of the users and the quality of image control [37,52].

## 6. OCT and OCTA Studies in Preclinical AD

The first study including patients with preclinical AD as participants was performed in patients with a family history of AD and memory complaints. In this cross-sectional study carried out with 63 participants, a non-significant thickening of the inner retinal plexiform layer (IPL) in the macular area of Aβ+ patients was observed in comparison to the control participants (Aβ−). In addition, the increase in the volume of this layer was associated with the surface area of the retinal inclusion bodies, which may contain deposits of fibrillar Aβ [53]. These inclusion bodies appear as faint white spots with poor border demarcation in SD-OCT imaging These inclusion bodies should not be confused with the drusen that occur in age-related macular degeneration and usually accumulate between Bruch’s membrane and the RPE [54,55,56]. In the RNFL, an increase in volume that was non-significant with respect to the Aβ-group was also observed. Furthermore, they studied the number and area of the surface inclusion retinal bodies, finding that their surface area increased as a consequence of the neocortical accumulation of Aβ. The authors suggest that the IPL volume increase may reflect an early inflammatory process related to the disruption of cholinergic transmission that is caused by Aβ deposition in the neocortex and/or may be the result of these inclusion bodies occupying space within (and adjacent to) this retinal layer [54]. Table 1 summarizes the characteristics of the included studies.

In a subsequent study conducted in a cohort of patients with biomarkers positive for Aβ and subjective memory complaints, the authors found no differences in RNFL thickness between people with preclinical AD and the healthy controls. They also found no significant differences in RNFL thickness between the groups, nor any association between retinal structural measurements and Aβ determination by PET in the neocortex [57].

In a transversal study where the preclinical AD group was compared with the control healthy group, a thinning of the macular RNFL (mRNFL) was found in the nasal sector in the preclinical AD group. Furthermore, while the inferior sector showed a decrease in thickness, the superior sector showed a thickening, both of which were not statistically significant. The predominant thinning of the mRNFL within the nasal sector is consistent with previous OCT studies in patients with established AD. The nasal fibers follow the papillomacular bundle, which has the highest energy requirement and is therefore more susceptible to degenerative damage [58]. In contrast, in a longitudinal study with a 27 month follow-up, a significant decrease in volume was found in the mRNFL, accompanied by a decrease in volume and thickness in the inner nuclear layer (INL) and outer nuclear layer (ONL) in the inferior sectors. While RNFL thinning is associated with neocortical Aβ accumulation, in preclinical AD, it does not yet relate to the subjects’ episodic memory performance or problem-solving ability. However, this axonal loss appears to be slightly related to decreased audiovisual integration, which has been proposed as a marker of mild cognitive impairment [20]. In another 22-month longitudinal study of 145 healthy monozygotic twins, no differences in retinal thickness changes were found between Aβ+ and Aβ− individuals. In addition, the authors reported that there was less thinning of the IPL over time with a greater bonding of Aβ in the PET, suggesting that this could be due to the presence of deposits or an inflammatory process [59].

In another study carried out with 165 participants, most of them monozygotic twins (75 twin pairs and 15 incomplete twin pairs), the authors reported no differences in total retinal thickness in the inner retinal layers in the macular area and peripapillary RNFL thickness (pRNFL) in the Aβ+ group with respect to the Aβ− participants. In addition, they found a positive correlation between the Aβ measurement on PET and the total macular thickness in the inner ring that was not statistically significant [22]. The same authors performed a study with OCTA in preclinical AD twins. The Aβ+ group showed a significant higher vessel density in comparison to Aβ− subjects in both the inner and outer macular rings and pRNFL, with ROC curves that demonstrated the high sensitivity and specificity of the analysis of vascular density in the peripapillary region, showing that this could be used as a reliable biomarker. However, the FAZ showed no significant differences between the study groups. The authors postulated that the increased vascular density could be due to an inflammatory state of the retina in the early stages of amyloid accumulation, as in the brain, the accumulation of Aβ is usually inflammatory in nature. If this also occurs in the retina at the same time, it may be that in the preclinical stage of the disease, the inflammatory reaction could lead to vasodilatation, allowing the microvessels to be detected by OCTA. After this phase, the accumulation of Aβ would continue to cause damage, leading to a decrease in vascular density, as has already been reported in patients with established AD. This study also found a positive association between Aβ cerebral deposition and vessel density for all regions of the retina, while Aβ binding on PET tended to be related to higher vascular density in the retinal inner ring. In addition, the correlations between OCTA vascular parameters between twin pairs were moderate to high, except for the vascular density around the optic nerve [60].

In contrast to these findings, in a case–control study involving 30 participants—14 with positive AD biomarkers and 16 participants without AD biomarkers—the authors reported increased FAZ in patients with positive biomarkers compared to participants without AD biomarkers. One possible explanation is that, in the brain, deposits of Aβ and collagen that accumulate within the capillaries cause cell apoptosis and retinal vessel closure during disease progression. Another possible explanation could be the accumulation of Aβ in the inner retinal layers. These findings suggest that, in individuals with preclinical AD, vascular and structural changes occur in the retina even before clinical signs of the disease appear [23].

In the last study performed with participants with preclinical AD where the Aβ_42_/Tau ratio in cerebrospinal fluid was analyzed, it was reported that there were no significant differences between groups in terms of RNFL, ganglion cell-inner plexiform layer complex (GC-IPL), or total macular thickness, according to SD-OCT. However, the retinal function showed statistical differences, with photopic negative response, as a measure of inner retinal function, being significantly diminished in the preclinical AD group in comparison to the control group. These results are consistent with previous work by the same group of patients with severe AD, where a more pronounced atrophy of the inner retinal layers affecting the GCL early in the disease was reported [61].

A recent study analyzed the structural differences that appear in the retina of subjects at high genetic risk of the development of sporadic AD using OCT. The authors reported a statistical thinning in different sectors of the mRNFL, IPL, INL, and OPL in participants with a family history and carriers of the ApoE ɛ4 allele in comparison to the control group (without a family history and non-carriers of the ApoE ɛ4 allele). This thinning may correspond to early degenerative changes in these layers. These structural changes have been reported in other studies that included participants with preclinical AD and subjective memory complaints. However, in the present study, both groups were made up of cognitively healthy participants with no memory problems, so the changes that appear could be related to very early stages of AD and should be followed to know if they evolve into an preclinical form of AD [24].

There is no consensus regarding the criteria for the inclusion of participants in preclinical AD studies. Although preclinical stages require the presence of at least one biomarker of AD pathology (either Tau pathology (CSF or PET Tau) or amyloid pathology (CSF Aβ_42_ or PET amyloid)) and the absence of clinical signs and symptoms [25], several studies have included participants with subjective memory complaints [20,54,57]. Patients with subjective memory complaints have no objective pathology but achieve worse scores on neuropsychological tests, have a higher incidence of developing future cognitive impairment, and show a pattern of hippocampal atrophy similar to that of amnestic mild cognitive impairment in MRI. In addition, they show an increased activation of cognitive tasks that may represent a compensation for the loss of function analyzed with functional MRI [62].

Another factor to take into account is the wide age range of the subjects. There are studies in which there is a variability of more than 30 years of age between participants from the same sample [60]. In addition, almost all participants are over 60 years old. Advanced age is the greatest risk factor for the development of AD, and after the age of 65, the risk of AD doubles every five years [63], so it is important that preclinical studies are conducted in younger participants, thus identifying which group of individuals could be candidates for early intervention. In parallel, more longitudinal studies are necessary (currently only two are available [20,59]), as these would also allow us to know the evolution of the participants as well as the reliability of OCT in detecting changes in preclinical AD stages.

The ophthalmological inclusion criteria in some studies are not very rigorous, allowing the inclusion of pathologies that may alter OCT measurements. Other pathologies such as hypertension and diabetes may alter OCTA results [57].

The methodological differences between studies make it difficult to define the characteristics and evolution of these patients. An example of this is the variety of ways in which the retina has been studied by OCT. Four studies have analyzed each retinal layer separately [20,22,24,54], while other authors have performed layer complexes or total retinal analysis. Retinal changes in AD can go undetected when the total retinal thickness is analyzed. This is because there are compensatory mechanisms between adjacent retinal layers that can mask both thinning and thickening when the total retinal volume is studied [64]. Therefore, a segmentation and analysis of each layer is necessary to know what is really happening in the preclinical stages of AD.

## 7. Conclusions

In conclusion, there are very few studies that analyze patients with preclinical AD and very few of a longitudinal nature. Moreover, these studies include participants with memory problems, at very advanced ages, and with ocular and systemic pathologies that could modify the results obtained in OCT and OCTA. Of these studies, only one assessed the genetic risk of the development of Alzheimer-type dementia, with age being one of the most important risk factors. The very low number of participants means that the preclinical AD group is very small, because only 20% of the population had these positive biomarkers. This means that the results found cannot be applied to a general population.

For all these reasons, it would be interesting in future research to increase the number of participants, be more rigorous in the inclusion criteria, include genetic risk parameters at earlier ages, and exclude ocular and systemic pathologies. Furthermore, it is necessary to carry out longitudinal studies with longer follow-up times to assess the evolution of these subjects. This could provide us, if the results were consistent in repeated studies with the same methodology, with an understanding of the value of the retinal changes observed by OCT/OCTA as possible reliable, cost-effective, and non-invasive biomarkers of preclinical AD.

## Figures and Tables

**Table 1:** Included studies that analyze preclinical AD participants using OCT and OCTA.

Study	Study-Design, Sample Size	Participant’s Status, (M/F), Years	Biomarker Preclinical AD	Neurological Test	OCT/OCT-A Model	OCT-OCT-A Parameters	Significant Parameters
Snyder et al., 2016	Cross-sectional (*n* = 63)	*n* = 10 Preclinical AD, (2/8), 62.28 (5.15) *n* = 53 Controls; (22/31), 65.50 (5.87)	Mean Aβ SUVr ratio 1.27 (0.22)	GDS DASS MAC-Q MMSE GMLT ISLT	Heidelberg Spectralis SD-OCT	pRNFL, mRNFL, GCL, IPL, INL, OPL, ONL	↑ IPL
Golzan et al., 2017	Cross-sectional (*n* = 73)	*n* = 50 Elderly control group, (14/36), 79 ± 5 *n* = 23 Preclinical AD group (9/14) 80 ± 4	Mean Aβ, SURVr 1.75 ± 0.24	MAC-Q MoCA	Nidek SD-OCT	pRNFL Macular RGCL complex	None
O’Bryhim et al., 2018	Observational case-control (*n* = 32)	*n* = 14 Preclinical AD. (8/6), 73.5 (4,7) *n* = 18 Control, 8/10, 75,2 (6,6)	Aβ+ CSF and/or PET (PiB or F-AV-45)	CDR	Optovue OCT-A System	Total and Temporal RNFL thickness; GCL thickness; Macular Volume; Inner, outer and total foveal thickness; total macular, foveal, paravofeal vascular density and FAZ	↑ FAZ
Santo et al., 2018	Longitudinal 27 months follow-up (*n* = 56)	*n* = 15 Preclinical AD, (4/11), 68.25 (5.81) *n* = 41 Controls, (17/24), 64.56 (5.26)	Mean Aβ SUVr ratio 1.32 (0.18)	MMSE MAC-Q ISLT GMLT	Heidelberg Spectralis SD-OCT	pRNFL, mRNFL, CGL, IPL, INL, OPL, ONL	↓ mRNFL ↓ INL and ONL inferior sector
van de Kreeke et al., 2019	Observational case-control (*n* = 124)	*n* = 13 preclinical AD *n* = 111 controls Overall (58/66), 66.6 ± 6.3	Global BP _ND_ of Aβ 0.122 (0.095–0.177)	MMSE	Zeiss Cirrus 5000 OCT-A	Retinal vessel density in inner and outer ring macula and around ONH. FAZ	↑ Retinal vessel density in inner and outer ring macula and around ONH
van de Kreeke et al., 2019	Cross-sectional (*n* = 165)	*n* = 18 Preclinical AD *n* = 147 control Overall (70/95), 69.5 (6.9)	Mean BPND of Aβ 0.120 (0.87–0.177)	MMSE	Heidelberg Spectralis SD-OCT	pRNFL, mRNFL, GCL, IPL	None
van de Kreeke et al., 2020	Longitudinal 22 months follow-up (*n* = 145)	*n* = 16 Preclinical AD *n* = 129 Controls Overall (67/78), 68.6 ± 6.3–70.5 ± 6.2	Mean BPND of Aβ 0.120 (0.088–0.174)	MMSE	Heidelberg Spectralis SD-OCT	pRNFL, mRNFL, GCL, IPL	None
López-Cuenca et al., 2020	Cross sectional (*n* = 64)	*n* = 35 FH+ Carriers of ApoE ε4, (11/24), 57.00(54.00–61.00) *n* = 29 FH-Non-Carriers of ApoE ε4, (12/17), 59.00 (54.00–65.00)	None	MMSE	Heidelberg Spectralis SD-OCT	pRNFL, Total retinal thickness, mRnfl, GCL, IPL, INL, OPL, ONL, RPE	↓ mRNFL central sector, ↓ IPL inferior and nasal sectors, ↓ INL central and inferior sectors, ↓ OPL inferior sector
Asanad et al., 2021	Cross sectional (*n* = 29)	*n* = 15 Preclinical AD, (3/12), 76.5 ± 6.6 *n* = 14 Controls (4/10), (79.9 ± 8.5)	Aβ42/Tau ratio in CSF (1.3 ± 0.4)	Uniform Data Set-3 criteria of the NACC	Zeiss Cirrus SD-OCT	RNFL, GCL-IPL, Total macular thickness	None

M, males; F, Females; OCT, Optical Coherence Tomography; OCT-A, Optical Coherence Tomography-Angiography, SD-OCT, Spectral Domain Optical Coherence Tomography; Aβ+, Aβ Amyloid positive; AD, Alzheimer’s disease; SUVR, Standardized Uptake Value Ratio; BPND, Non-displaceable Binding Potential; CSF, Cerebrospinal Fluid, GDS, Geriatric Depression Scale; DASS, Depression, Anxiety, and Stress Scale; MAC-Q, Memory Complaints Questionnaire; MMSE, Mini Mental State Examination; GMLT, Groton Maze Learning Test; ISLT, International Shopping List Task; MoCA, Montreal Cognitive Assessment, CDR, Clinical Dementia Rating; NACC, National Alzheimer’s Coordinating Center; FH+, subjects with a family history of AD; ApoE, Apolipoprotein E; FH-, subject without a family history of AD; pRNFL, Peripapillary Retinal Nerve Fiber Layer; mRNFL, Macular Retinal Nerve Fiber Layer; GCL, Ganglion Cell Layer; IPL, Inner Plexiform Layer; INL, Inner Nuclear Layer; OPL; Outer Plexiform Layer; ONL, Outer Nuclear Layer; RPE, Retinal Pigment Epithelium.

## Data Availability

Not applicable.
